# Kinematic differences between hitting off a tee versus front toss in collegiate softball players

**DOI:** 10.1080/23335432.2018.1472038

**Published:** 2018-05-15

**Authors:** Jessica Washington, Gretchen Oliver

**Affiliations:** a School of Kinesiology, Auburn University, Auburn, AL USA

**Keywords:** Batting, biomechanics, movement coordination, performance

## Abstract

The purpose of this study was to compare kinematics of two hitting conditions: stationary tee and front toss from a practice pitcher. Twenty-two NCAA Division I Collegiate softball players (20.3 ± 1.5 years; 166.6 ± 6.3 cm; 68.0 ± 7.5 kg) participated. Participants executed five maximum effort swings from a stationary tee and five swings from a front toss practice pitcher. Data for each kinematic variable were averaged for the five maximal effort swings of each condition and analyzed using a within-subject repeated measures ANOVA. The front toss condition revealed significantly greater lead knee flexion at foot contact and greater trunk rotation towards the back side at ball contact. The tee condition revealed greater trunk lateral flexion to the back side at foot contact, greater trunk rotation towards the lead side at follow through, and greater pelvis rotation towards the lead side at follow through. This study most significantly indicates that swing mechanics are different between specific training methods; therefore, athletes should implement techniques most applicable to a competition setting such as the front toss.

## Introduction

Hitting a baseball or softball is speculated to be one of the most complex skills in sport (Williams and Underwood 1986; DeRenne 2007; Escamilla et al. 2009). Development of proper hitting mechanics often begins at a young age and utilizes basic training techniques, including a stationary hitting tee and front toss from a practice pitcher. While the stationary tee is most commonly viewed as method for establishing fundamental hitting mechanics, elite athletes use this tool to maintain proper mechanics in their daily training regimen, as well (Winkin et al. 2001). Front toss is the practice tool most similar to a game setting, requiring the athlete to not only focus on their swing mechanics but also on the location and velocity of the ball thrown, thereby most effectively preparing an athlete for competition (Winkin et al. 2001).

Previously, kinematics and kinetics of hitting from a tee as well as front toss have been individually examined in baseball and softball athletes (Katsumata 2007; Inkster et al. 2010; Fleisig et al. 2013; Lino et al. 2014; Ae et al. 2016; Dowling and Fleisig 2016). Baseball players of high skill level were found to have significantly higher lead elbow angular velocity and hip rotation velocity compared to their counterparts of lower skill level (Inkster et al. 2010). At ball contact, adult baseball hitters exhibit significantly greater back shoulder abduction and less back elbow flexion, indicating that they hold the bat further away from the body at this instant (Dowling and Fleisig 2016). In examination of kinetics, it was found that elite baseball athletes exhibit different upper extremity torques when hitting pitches of varying height (Ae et al. 2016), and, among softball athletes, pelvis rotation torque prior to ball contact also differs as a function of pitch height (Lino et al. 2014). Therefore, it can be determined that certain factors, namely skill level, age, and pitch location, do influence hitting kinematics and kinetics in baseball and softball athletes.

While valuable insight into mechanical differences between age groups, athletes of varying skill and the influence of pitch variation were acquired from these data, each of these studies implemented one condition, either the stationary tee or use of a practice pitcher, not both. To the authors’ knowledge, there has yet to be any comparison of swing mechanics between hitting from a tee versus front toss. As the goal of basic training methods is to establish proper fundamental mechanics and successful transfer of these mechanics to a competition setting, kinematic comparisons of the stationary tee and front toss are essential in understanding whether basic training methods do appropriately prepare an athlete for competition. While it is argued that the baseball and softball swing are very similar, limited data exist on the kinematics and kinetics of either. Therefore, the purpose of this study was to compare lower extremity and trunk kinematics (lead knee flexion, pelvis rotation, trunk rotation, trunk lateral flexion, and trunk flexion) between two common hitting conditions: a stationary tee and front toss from a practice pitcher. It was hypothesized that significant differences in mechanics at the lead knee, pelvis, and trunk would be observed between conditions. Specifically, the front toss condition would result in increased lead knee flexion, pelvis rotation towards the lead side, trunk rotation towards the lead side, trunk lateral flexion towards the back side, and trunk flexion as compared to the stationary tee condition, because it is known that variances in ball height and velocity can result in different swing mechanics at these segments (Katsumata 2007; Lino et al. 2014; Ae et al. 2016).

## Methods

### Participants

Twenty-two NCAA Division I Collegiate softball players (20.3 ± 1.5 years; 166.6 ± 6.3 cm; 68.0 ± 7.5 kg) participated in the study. Seven participants hit left-handed, while the remaining 15 hit right-handed. Selection criteria included being currently active on the playing roster and medically cleared by all sports medicine staff. Potential participants with a history of lower extremity injury within the past six months were excluded. Auburn University’s Institutional Review Board approved all testing protocols. Prior to data collection, all testing procedures were explained to each participant and informed consent was obtained. All data were collected in the Sports Medicine and Movement Laboratory using a standard 70 foot batting cage to most accurately reflect a hitter’s training setting and provide the ability to move freely without restriction while hitting.

### Procedures

**Figure 1.**
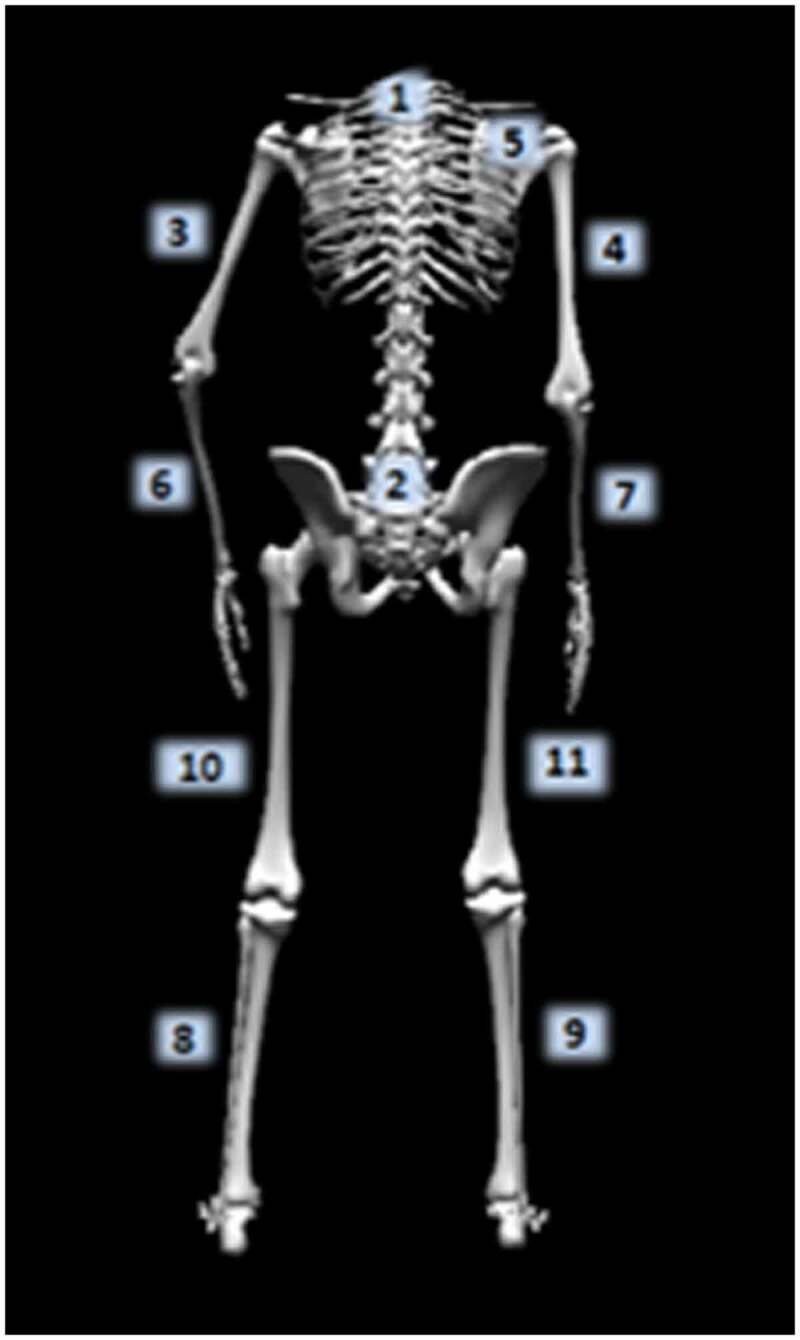
Electromagnetic sensor assignments

All kinematic data were collected with The MotionMonitor^TM^ (Innovative Sports Training, Chicago, IL) synchronized with an electromagnetic tracking system (Track Star, Ascension Technologies Inc., Burlington, VT). Field distortion associated with electromagnetic tracking systems was previously reported to cause errors greater than 5° at a distance of two meters from the extended range transmitter, but instrument sensitivity increases have reduced this error from near 10° prior to system calibration to 2° following calibration (Meskers et al. 1999; Day et al. 2000; Perie et al. 2002). The system was calibrated using previously established protocols prior to the collection of any data (Day et al. 2000; Oliver and Keeley 2010; Plummer and Oliver 2014). After calibration, the error in determining position and orientation of the electromagnetic sensors was less than 0.01 m and 2°, respectively. Intra-rater reliability of digitization was determined during a pilot study of 9 collegiate softball athletes. The investigator reported an intra-rater reliability of an ICC(3,k) of 0.75–0.93 for all digitization measurements. Eleven electromagnetic sensors were attached to the following locations: (1) the posterior/medial aspect of the torso at T1, (2) posterior/medial aspect of the pelvis at S1, (3–4) bilateral distal/posterior aspect of the upper arm, (5) the flat, broad portion of the acromion of the scapula, (6–7) bilateral distal/posterior aspect of the forearm, (8–9) bilateral distal/lateral aspect of the lower leg, and (10–11) bilateral distal/lateral aspect of the upper leg (Figure 1) (Myers et al. 2005, 2013; Oliver and Keeley 2010; Oliver 2014; Oliver and Plummer 2015; Plummer and Oliver 2016).

**Figure 2.**
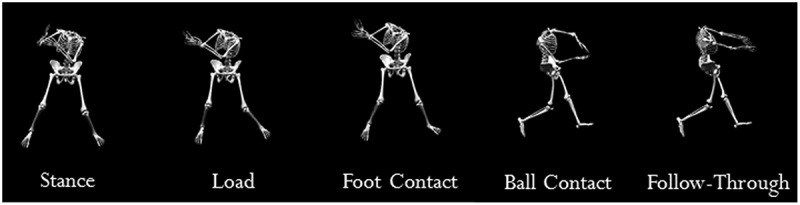
Hitting motion events

Notes: ST = stance; LD = load; FC = stride foot contact; BC = ball contact; FT = follow through.

Once all sensors were secured, participants were given verbal instruction to perform their own specified warm-up (average warm-up time was 5 min). Warm-up was not standardized across participants as some hitters needed more time than others to feel sufficiently warm and capable of executing maximum effort swings without risking injury. Each participant used her personal softball bat to reduce variability otherwise accrued due to adaption of unfamiliar equipment. Participants were instructed to execute five maximum effort swings from of a stationary tee and five maximal effort swings from a front toss practice pitcher located 9.14 m away. Condition performed first was randomized for each participant such that every participant did not execute stationary tee swings prior to front toss swings. Stationary tee height was determined as a function of participant height. The tee was placed approximately midway between the knee and hip and located at a distance away from the body to reflect a pitch thrown over the middle of the strike zone. One front toss practice pitcher was used throughout collection to minimize increased variability due to multiple pitchers. Average pitch velocity was 20.1 m/s. Saved trial criteria included (1) result of a line drive from the tee or front toss practice pitcher; (2) pitch location over the middle of the strike zone (i.e. middle of home plate and midway between the hitter’s knee and hip) from the front toss practice pitcher; and (3) verbal approval by the hitter as a ‘good’ swing. Approval from the participant was desired, because the softball swing varies from athlete to athlete and because the temporal and ‘feel’ components of a hitter’s swing has been reported as essential to a successful performance outcome (Williams and Underwood 1986). Data for each kinematic variable were averaged for the five maximal effort swings in an effort to limit potential variability between trials. The hitting movement was divided into the events of stance, load, stride foot contact, ball contact, and follow through. All kinematic variables were analyzed at these events (Figure 2) (Dowling and Fleisig 2016).

Medial and lateral aspects of each joint were identified and digitized. Joint centers were calculated by the midpoint of the two points digitized. A link segment model was then developed through digitization of bony landmarks used to estimate the joint centers for the ankle, knee, thoracic vertebrae 12 (T12) to lumbar vertebrae 1 (L1), and cervical vertebrae 7 (C7) to thoracic vertebrae 1 (T1). The spinal column was defined as the digitized space between the associated spinous processes, whereas the ankle and knee were defined as the midpoints of the digitized medial and lateral malleoli, and the medial and lateral femoral condyles, respectively (Wu et al. 2002; Oliver and Keeley 2010; Oliver and Plummer 2011, 2015; Oliver 2014).

The shoulder and hip joint centers were estimated using the rotation method as it has been shown to provide accurate positional data (Veeger 2000). The shoulder joint center was calculated from the rotation between the humerus relative to the scapula, and the hip joint center was calculated from the rotation of the femur relative to the pelvis. The point on the humerus or femur that moved the least according to a least-squares algorithm allowed for the calculation of the joint centers. The variation in the measurement of the joint center had to have a root mean square error of less than 0.001 m to be accepted. All kinematic data were sampled at a frequency of 100 Hz (Oliver 2014; Plummer and Oliver 2014; Oliver and Plummer 2015). Raw data regarding sensor orientation and position were transformed to locally based coordinate systems for each respective body segment. Two points described the longitudinal axis of each segment and the third point defined the plane of the segment. A second axis was defined perpendicular to the plane and the third axis was defined as perpendicular to the first and second axes. Neutral stance was the *y*-axis in the vertical direction, anterior/posterior of *y* in the direction of movement was the positive *x*-axis, and orthogonal to *x*-*y* axis and to the right was the positive z-axis. Pelvis, torso, and upper extremity kinematics were defined by the standards and conventions of The International Shoulder Group and International Society of Biomechanics (Wu et al. 2002, 2005).

### Statistical analysis

The independent variable in this study was hitting condition (tee versus front toss) while the dependent variables were lower extremity and trunk kinematics: lead knee flexion, pelvis rotation, trunk rotation, trunk lateral flexion, and trunk flexion. Data were analyzed using IBM SPSS Statistics 23 (IBM corp., Armonk, NY). Because swing mechanics vary by participant, a within-subject analysis was implemented in order to eliminate comparison variability between conditions. A 2 (condition) x 5 (hitting event) within-subject repeated measures ANOVA was utilized. If Mauchly’s Test of Sphericity was violated, a Greenhouse–Geisser correction statistic was reported. To determine kinematic differences in condition, post hoc simple effects tests were run for any statistically significant main effect or interaction that included the condition variable. However, if significant interactions were observed, main effects of condition were negated. For the purpose of analysis, all data were normalized to a right-handed hitting position. The left side of the body was defined as the lead side, while the right side was defined as the back side during the hitting motion. The alpha level was set a priori at *p* ≤ 0.05.

## Results

Differences in swing mechanics were observed at the lead knee, pelvis, and trunk (Table 1). A 2 (condition) × 5 (hitting event) within-subject repeated measures ANOVA resulted in a statistically significant condition by event interaction for lead knee flexion (*F*_4,84_ = 2.584, *p* = 0.043, $\eta_{p}^{2}$ = 0.110), pelvis rotation (*F*_4,84_ = 3.393, *p* = 0.031, $\eta_{p}^{2}$ = 0.139), trunk lateral flexion (*F*_4,8q4_ = 7.992, *p* = 0.001, $\eta_{p}^{2}$ = 0.276), and trunk rotation (*F*_4,84_ = 11.369, *p* < 0.001, $\eta_{p}^{2}$ = 0.351). However, trunk flexion resulted in a non-statistically significant condition by event interaction (*F*_4,84_ = 2.948, *p* = 0.053, $\eta_{p}^{2}$ = 0.123).

**Table 1.** Lower extremity and trunk kinematics throughout the hitting motion

	Tee	Toss
	Mean, SD	Mean, SD
*Stance (ST)*		
Lead Knee Flexion (°)	43.826 ± 12.798	42.428 ± 11.342
Pelvis Rotation (°)	−92.852 ± 13.191	−91.265 ± 14.700
Trunk Flexion (°)	−5.941 ± 9.598	−4.565 ± 9.927
Trunk Lateral Flexion (°)	17.260 ± 7.463	18.454 ± 7.548
Trunk Rotation (°)	−95.032 ± 25.194	−93.208 ± 25.280
*Load (LD)*		
Lead Knee Flexion (°)	53.159 ± 18.018	53.787 ± 17.238
Pelvis Rotation (°)	−93.927 ± 13.728	−92.716 ± 15.701
Trunk Flexion (°)	−6.854 ± 11.358	−4.289 ± 11.629
Trunk Lateral Flexion (°)	20.526 ± 8.307	20.189 ± 7.927
Trunk Rotation (°)	−98.857 ± 26.095	−96.671 ± 26.422
*Foot Contact (FC)*		
Lead Knee Flexion (°)	**35.442** ± **10.194** **	**38.067** ± **10.234** **
Pelvis Rotation (°)	−101.718 ± 7.941	−102.898 ± 10.448
Trunk Flexion (°)	0.976 ± 11.350	4.779 ± 11.658
Trunk Lateral Flexion (°)	**24.594** ± **8.395** **	**20.823** ± **8.283** **
Trunk Rotation (°)	−115.540 ± 26.881	−112.437 ± 23.518
*Ball Contact (BC)*		
Lead Knee Flexion (°)	17.550 ± 12.593	18.223 ± 13.083
Pelvis Rotation (°)	−12.017 ± 11.028	−14.200 ± 9.234
Trunk Flexion (°)	11.773 ± 9.596	12.638 ± 8.761
Trunk Lateral Flexion (°)	35.472 ± 7.708	35.813 ± 8.028
Trunk Rotation (°)	**−23.924** ± **29.572** **	**−30.068** ± **26.457** **
*Follow Through (FT)*		
Lead Knee Flexion (°)	6.031 ± 12.162	4.264 ± 11.712
Pelvis Rotation (°)	**2.115** ± **9.878** **	**0.063** ± **9.454** **
Trunk Flexion (°)	6.820 ± 8.778	6.997 ± 7.840
Trunk Lateral Flexion (°)	38.739 ± 8.334	38.712 ± 8.264
Trunk Rotation (°)	**20.383** ± **24.207** **	**13.902** ± **22.908** **

Notes: Pelvis rotation: (+) towards lead side, (−) towards back side; Trunk flexion: (+) flexion, (−) extension; Trunk lateral flexion: (+) towards back side, (−) towards lead side; Trunk rotation: (+) towards lead side, (−) towards back side.

** Significant differences between stationary tee and front toss conditions. *p* ≤ 0.05.

Kinematics at the lead knee were the only expected result as post hoc simple effects tests revealed significantly greater lead knee flexion during the front toss condition at foot contact (mean difference = 2.625; 95% CI = 0.564, 4.686; *p* = 0.015) (Table 2). Post hoc simple effects tests revealed surprising differences at the trunk and pelvis between conditions, with most differences resulting in a more exaggerated position at crucial events in the swing during the tee condition rather than the front toss condition (Table 2). Greater trunk lateral flexion to the back side at foot contact during the tee condition (mean difference = 3.771; 95% CI = 2.232, 5.311; *p* < 0.001), greater trunk rotation towards the back side at ball contact during front toss (mean difference = 6.14; 95% CI = 1.02, 11.27; *p* = .021), greater trunk rotation towards the lead side at follow through during the tee condition (mean difference = 6.48; 95% CI = 1.26, 11.70; *p* = .017), and greater pelvis rotation towards the lead side at follow through during the tee condition (mean difference = 2.05; 95% CI = 0.17, 3.94; *p* = .034) were observed (Table 2).

**Table 2.** Post hoc simple effects tests of pairwise comparisons in lower extremity and trunk kinematics throughout the hitting motion

	Tee versus Toss
	*p-*value (95% CI)
*Stance (ST)*	
Lead Knee Flexion (°)	0.306 (−4.167, 1.371)
Pelvis Rotation (°)	0.101 (−3.509, 0.336)
Trunk Flexion (°)	–
Trunk Lateral Flexion (°)	0.088 (−2.583, 0.194)
Trunk Rotation (°)	0.065 (−3.771, 0.124)
*Load (LD)*	
Lead Knee Flexion (°)	0.697 (−2.682, 3.937)
Pelvis Rotation (°)	0.285 (−3.506, 1.084)
Trunk Flexion (°)	–
Trunk Lateral Flexion (°)	0.581 (−0.915, 1.590)
Trunk Rotation (°)	0.082 (−4.671, 0.300)
*Foot Contact (FC)*	
Lead Knee Flexion (°)	**0.015 (0.564, 4.686)** **
Pelvis Rotation (°)	0.436 (−1.911, 4.270)
Trunk Flexion (°)	–
Trunk Lateral Flexion (°)	**<0.001 (2.232, 5.311)** **
Trunk Rotation (°)	0.071 (−6.497, 0.290)
*Ball Contact (BC)*	
Lead Knee Flexion (°)	0.483 (−1.286, 2.633)
Pelvis Rotation (°)	0.154 (−0.883, 5.251)
Trunk Flexion (°)	–
Trunk Lateral Flexion (°)	0.654 (−1.898, 1.217)
Trunk Rotation (°)	**0.021 (1.020, 11.267)** **
*Follow Through (FT)*	
Lead Knee Flexion (°)	0.084 (−3.794, 0.261)
Pelvis Rotation (°)	**0.034 (0.166, 3.938)** **
Trunk Flexion (°)	–
Trunk Lateral Flexion (°)	0.970 (−1.448, 1.502)
Trunk Rotation (°)	**0.017 (1.261, 11.701)** **

** Statistically significant difference in condition. *p* ≤ 0.05.

## Discussion

The purpose of this study was to compare lower extremity and trunk kinematics (lead knee flexion, pelvis rotation, trunk rotation, trunk lateral flexion, and trunk flexion) between two hitting conditions (stationary tee and front toss). It was hypothesized that significant differences in mechanics at the lead knee, pelvis, and trunk would be observed. Specifically, the front toss condition would result in increased lead knee flexion, pelvis rotation towards the lead side, trunk rotation towards the lead side, trunk lateral flexion towards the back side, and trunk flexion as compared to the stationary tee condition as it is known that variances in ball height and velocity can result in different swing mechanics at these segments (Katsumata 2007; Lino et al. 2014; Ae et al. 2016). The observed results reject the null hypothesis in that the examined participants displayed different positions at specific events throughout the hitting motion, during both the tee and front toss condition.

At foot contact, participants in the current study displayed greater lead knee flexion during the front toss condition and greater trunk lateral flexion towards the back side during the tee condition. The increase in knee flexion during the front toss can most likely be attributed to the variation in pitch location under this condition compared to a stationary ball under the tee condition. A more lateral trunk position at foot contact indicates an inherent preparatory exaggeration prior to hitting a stationary ball. Because front toss mitigates more focus on the pitch velocity and location, whereas hitting from a stationary tee does not, hitters may unintentionally overstress their preparation in order to ultimately hit the ball with more force resulting in a longer hit. While a more desirable hit outcome may result from these mechanics, caution should be taken as the tee condition does not more accurately reflect a competition setting, such as the front toss condition. Literature has shown in right handed hitters that the right external oblique muscle fires 0.02 s prior to the left external oblique in a hitting motion from a stationary tee (Lim et al. 2016). Lateral trunk flexion to the back side as a preparatory movement could cause the left external oblique to fire early in attempt to stabilize the trunk, resulting in a less efficient firing pattern during rotation to ball contact. Thus, further investigation is necessary to determine whether these alternate preparation mechanics and firing patterns are more accurate and more efficient in a hitter’s training.

The participants in the current study exhibited greater trunk rotation toward the back side at ball contact during the front toss condition. This position could explain speculation of hitters allowing the pitch to travel further towards the plate prior to initiating the swing, resulting in a different timing pattern between a stationary tee and live pitcher conditions. The latency in initiating trunk rotation in the front toss condition may provide better efficiency in utilizing the potential energy stored in the trunk rotator musculature. Allowing the pitch to travel further also allows the hitter to better read the pitch location and velocity prior to swing initiation which would ultimately lead to a more desirable outcome as the hitter would have more knowledge on where to swing the bat through the ‘strike zone’. However, investigation into swing timing and the differences in sequence efficiency based on this timing is needed to support this notion.

At follow through, participants displayed greater pelvis and trunk rotation towards the lead side during the tee condition. This result could again be the product of purposeful exaggeration in mechanics during the tee condition. Since the ball is stationary, focus of ball location and velocity from a pitcher’s hand is eliminated theoretically increasing body awareness thereby encouraging the hitter to emphasize swing mechanics for a better swing outcome. Furthermore, a strong follow through is typically emphasized in a training program to encourage the hitter to finish the swing (Williams and Underwood 1986). Focus on a strong finish could theoretically result in more force behind the ball and ultimately a further ball trajectory, especially in a competition setting (Williams and Underwood 1986). However, these results suggest that focus on following through the swing may be decreased in a competitive setting and thus should be of emphasis in specific training protocols.

While these results may provide valuable information into training protocols used in softball and baseball hitting alike, limitations in this study do exist. All participants were members of the same team, thus, all participants completed the same training regimen. It should, however, be noted that each participant executes swings from a stationary tee and front toss daily, especially during competition season when most of these data were collected. Additionally, because the front toss condition pitch locations were deemed accurate through visual observation, variability caused by pitch location during this condition is increased and, therefore, may be considered speculative. Lastly, variation in warm-up time could ultimately affect swing mechanics as some athletes were looser than others.

## Conclusion

This study most significantly indicates that swing mechanics are different between two common training methods in hitting: the stationary tee and front toss from a practice pitcher. As most differences occurred at the trunk during all active phases of the swing, emphasis should be placed on transfer of proper mechanics in the mid-region between training and competition. As the trunk assists in stabilization of the upper extremity, proper mechanics in the trunk could lead to improved swing outcomes.
